# The influence of fear of falling on gait variability: results from a large elderly population-based cross-sectional study

**DOI:** 10.1186/1743-0003-11-128

**Published:** 2014-08-29

**Authors:** Farah Ayoubi, Cyrille P Launay, Anastasiia Kabeshova, Bruno Fantino, Cédric Annweiler, Olivier Beauchet

**Affiliations:** Department of Neuroscience, Division of Geriatric Medicine, Angers University Hospital, Angers, 49933 France; UPRES EA 4638, Angers University Hospita, Angers, France; Robarts Research Institute, Department of Medical Biophysics, Schulich School of Medicine and Dentistry, the University of Western Ontario, London, Ontario Canada

**Keywords:** Fear, Accidental falls, Gait disorders, Aged

## Abstract

**Objective:**

To compare gait variability among older community-dwellers with and without fear of falling and history of falls, and 2) to examine the association between gait variability and fear of falling while taking into account the effect of potential confounders.

**Methods:**

Based on a cross-sectional design, 1,023 French community-dwellers (mean age ± SD, 70.5 ± 5.0 years; 50.7% women) were included in this study. The primary endpoints were fear of falling, stride-to-stride variability of stride time and walking speed measured using GAITRite® system. Age, gender, history of falls, number of drugs daily taken per day, body mass index, lower-limb proprioception, visual acuity, use of psychoactive drugs and cognitive impairment were used as covariables in the statistical analysis. P-values less than 0.05 were considered as statistically significant.

**Results:**

A total of 60.5% (n = 619) participants were non-fallers without fear of falling, 19% (n = 194) fallers without fear of falling, 9.9% (n = 101) non-fallers with fear of falling, and 10.7% (n = 109) fallers with fear of falling. Stride-to-stride variability of stride time was significantly higher in fallers with fear of falling compared to non-fallers without fear of falling. Full adjusted linear regression models showed that only lower walking speed value was associated to an increase in stride-to-stride variability of stride time and not fear of falling, falls or their combination. While using a walking speed ≥1.14 m/s (i.e., level of walking speed that did not influence stride-to-stride variability of stride time), age and combination of fear of falling with history of previous falls were significantly associated with an increased stride-to-stride variability of stride time.

**Conclusions:**

The findings show that the combination of fear of falling with falls increased stride-to-stride variability of stride time. However, the effect of this combination depended on the level of walking speed, increase in stride-to-stride variability of stride time at lower walking speed being related to a biomechanical effect overriding fear of falling-related effects.

**Electronic supplementary material:**

The online version of this article (doi:10.1186/1743-0003-11-128) contains supplementary material, which is available to authorized users.

## Background

Fear of falling (FOF), gait impairment and falls are common in older adults with a high prevalence estimated over 20%[1, 2]. They share the same risk factors such age, depressive mood and cognitive decline, as well as adverse consequences including activity restriction, increase in frailty and decrease in quality of life[3, 4]. Because of a complex interplay between FOF, gait impairment and falls, less is known on the causal relationship between FOF and gait impairment in older adults[5]. A better understanding on the association of FOF with gait impairment in aging population may be useful to appreciate the interaction between FOF and age-related changes in gait control, and to implement efficient prevention strategies of FOF.

Gait impairment has been reported among older individuals with FOF[6–9]. Most of previous studies used mean values of spatial-temporal gait parameters and reported low gait performance including slower walking speed, shorter stride length and increased double support or stride width[6, 7]. FOF-related changes in gait performance are usually classified as higher-level gait disorders[8, 9]. It is now well established that gait variability defined as fluctuation of spatial-temporal gait parameters with time, is a biomarker of higher-level gait disorders[9, 10]. In particular, stride-to-stride variability of stride time (STV) - a measure of the reliability of lower limb movements - has been identified as a dependable biomarker of the rhythmic stepping mechanism depending on the highest-levels of gait control[10]. Higher STV reflects an inefficient gait control and, thus, an unsafe gait[11].

A limited number of studies have examined the association between FOF and higher STV, and have showed mixed results. Indeed, although some reported an association, others did not[2, 5, 8, 9]. We suggest that divergences previously reported are related to the effect of main confounders previously identified in the literature that may increase STV independently of FOF such as the age, history of falls, cognitive decline and low walking speed[10, 12]. Thus, the question is to determine whether FOF may independently influence or not gait variability among older adults. The aims of our study were 1) to compare STV among of older community-dwellers with and without FOF and falls, and 2) to examine the association between STV and FOF while taking into account the effect of some known potential confounders including walking speed.

## Methods

### Population and study design

Between July 17^th^ 2008 and April 3^rd^ 2012, 1,023 community-dwellers were recruited in the French health examination center (HEC) of Lyon, localized in Eastern France, during a free medical examination. Exclusion criteria for the present analysis included age below 65 years, institutionalization, inability to understand and speak French, acute medical illness during the past month, diagnosis of dementia, extrapyramidal rigidity of the upper limbs (score above 2 on item no. 22 of the Unified Parkinson’s Disease Rating Scale motor score)[13], severe orthopaedic diagnoses involving the lumber vertebra, pelvis or lower extremities (i.e., severe osteoarthritis and prosthesis) and inability to walk 6 meters unassisted.

### Clinical assessment

Baseline assessment included a full medical examination including information on age, gender and measures of height and weight. Body mass index (BMI, in kg/m^2^) was calculated based on anthropometry measurements (i.e., weight in kg and height in m). The number of drugs daily taken and the use of psychoactive drugs including benzodiazepines, antidepressants or neuroleptics, were also recorded. FOF was assessed using a single question: "Are you afraid of falling?" with a binary answer (i.e., Yes versus No). History of falls over the past year was recorded using a standardized questionnaire based on 22 items exploring the number, delay and place of falls (i.e., inside or outside the participant’s house), the evoked causes and circumstances of falls and all physical traumatisms[14]. A fall was defined as unintentionally coming to rest on the ground, floor, or other lower level and not as the result of a major intrinsic event or an overwhelming hazard[15]. Thus, falls resulting from acute medical events and/or external force were excluded from the analysis. Fallers were defined by the occurrence of at least one fall during the last year[16]. Lower limb proprioception was evaluated with a 64 Hz graduated tuning fork placed on the tibial tuberosity[17]. The mean value obtained for the left and right sides was used in the present data analysis. Distance binocular vision was measured at 5 m with a standard Monoyer letter chart[18]. Vision was assessed with corrective lenses on if used by the participant. Depression was evaluated with the use of the 4-item Geriatric depression scale (GDS) score[19]. A score ≥1 indicated the presence of depressive symptoms. Cognitive decline, and more precisely executive dysfunction, was considered when the clock drawing test was abnormal (i.e., one or more errors were made in the execution of drawing the face of the clock and/or the hands of the clock)[20].

### Gait recording

STV and walking speed were measured at self-selected waking speed using GAITRite®-system (GAITRite Gold, CIR Systems, PA, USA) in a 6-meter corridor. The GAITRite®-System is an electronic walkway-integrated, pressure-sensitive electronic surface of 5.6 x 0.89 m that is connected to a personal portable computer via an interface cable. Participants walked one trial at their usual self-selected walking speed in a quiet, well-lit environment wearing their own footwear according to European guidelines for spatio-temporal gait analysis in older adults[21]. Coefficient of variation (CoV) (CoV = (standard deviation / mean) x 100) of stride time was used to explore the outcome measure of STV.

### Standard protocol approvals, registrations, and patient consents

Participant in the study were included after having given their written informed consent for research. The study was conducted in accordance with the ethical standards set forth in the Helsinki Declaration (1983). The entire study protocol was approved by the local Ethical Committee of Lyon (France) and the study is in compliance with the STROBE statement guidelines.

### Statistical analysis

The participants’ characteristics were summarized using means and standard deviations or frequencies and percentages, as appropriate. Normality of data distribution was checked using skewness-kurtosis test. As the number of observations was > 40 for each group, no transformations were applied to the variables of interest. For the current analysis, participants were classified into 4 groups as follows: No FOF and no falls; no FOF and falls; FOF and no falls; FOF and falls. First, between-group comparisons were performed using one-way analysis of variance (ANOVA) with Bonferroni corrections or Chi-square test, as appropriate. Second, univariate and multiple linear regression analyses were performed to examine the association between CoV of stride time (dependent variable) and FOF (independent variable) adjusted on walking speed and participants’ baseline characteristics. Third, a logarithmic regression of the association between STV and walking speed of participants separated into four groups based on FOF and history of falls was performed to identify threshold value of walking related to increase in STV. P-values less than 0.05 were considered as statistically significant. All statistics were performed using SPSS (version 19.0; SPSS, Inc., Chicago, IL, USA).

## Results

Among 1,023 included participants, 60.5% (n = 619) were non-fallers without FOF, 19% (n = 194) fallers without FOF, 9.9% (n = 101) non-fallers with FOF, and 10.7% (n = 109) fallers with FOF. As shown in Table 1, the prevalence of women and the number of drugs daily taken were higher among participants with FOF compared to those without FOF (P < 0.025), regardless of the history of previous falls. Participants with FOF walked slower than those without FOF (P < 0.008). STV was significantly higher in fallers with FOF compared to non-fallers without FOF (P = 0.003).

**Table 1:** **Comparisons of the participants’ characteristics separated into four groups based on fear of falling and history of falls (n = 1023)**

	Total (n = 1023)	Fear of falling				P-value*						
		No (n = 813)		Yes (n = 210)								
		≥ 1 fall		≥ 1 fall								
		No	Yes	No	Yes	Overall	G1 versus G2	G1 versus G3	G1 versus G4	G2 versus G3	G2 versus G4	G3 vs G4
		(n = 619)	(n = 194)	(n = 101)	(n = 109)							
		G1	G2	G3	G4							
Age (years), mean ± SD	70.5 ± 5	70.3 ± 4.8	70.5 ± 5.0	70.8 ± 5.5	71 ± 5.2	0.634	1.000	1.000	1.000	1.000	1.000	1.000
Female gender, n (%)	519 (50.7)	251 (40.5)	111 (57.2)	72 (71.3)	85 (78)	<0.001	<0.001	<0.001	<0.001	0.104	0.002	1.000
Number of drugs taken per day, mean ± SD	3.0 ± 2.4	2.6 ± 2.3	2.8 ± 2.3	3.7 ± 2.6	4.2 ± 2.7	<0.001	1.000	<0.001	<0.001	0.024	<0.001	1.000
Body mass index (kg/m2), mean ± SD	26.3 ± 4	26.1 ± 3.8	26.4 ± 3.9	26.4 ± 4.6	27.1 ± 5.1	0.111	1.000	1.000	0.095	1.000	0.734	1.000
Lower limb proprioception† (/8), mean ± SD	6.4 ± 1.9	6.5 ± 1.8	6.5 ± 1.9	6.3 ± 2.1	6.1 ± 2.0	0.236	1.000	1.000	0.431	1.000	0.464	1.000
Visual acuity‡ (/10), mean ± SD	6.9 ± 2.1	7.0 ± 2.1	6.7 ± 2.1	6.8 ± 2.1	6.6 ± 2.2	0.178	0.855	1.000	0.371	1.000	1.000	1.000
Walking speed (cm/s), mean ± SD	107.7 ± 22.6	110.8 ± 21.5	107.0 ± 21.8	103.0 ± 25.2	96.17 ± 23.1	<0.001	0.226	0.007	<0.001	0.870	<0.001	0.154
Use of psychoactive drugs¶, n (%)	185 (18.1)	84 (13.6)	30 (15.5)	34 (33.7)	37 (33.9	0.140	0.583	0.772	1.000	1.000	0.565	0.603
Cognitive impairment||, n (%)	213 (20.8)	122 (18.7)	39 (20.1)	23 (22.8)	29 (26.6)	0.397	1.000	1.000	0.615	1.000	1.000	1.000
Stride-to-stride variability of stride time (%), mean ± SD	2.0 ± 2.6	2.0 ± 2.1	2.0 ± 2.5	3.0 ± 4.5	3.0 ± 2.8	0.002	1.000	0.225	0.003	0.873	0.053	1.000

G1: Non-fallers and no fear of falling; G2: Fallers and no fear of falling; G3: Non-Fallers and fear of falling; G4: Fallers and fear of falling.

*: Comparison based on oneway ANOVA with Bonferroni corrections or Chi-square test, as appropriate.

†: Mean value of left and right side and based on graduated tuning fork placed on the lower limb.

‡: Binocular visual acuity at a distance of 5 m with a Snellen letter test chart.

¶: Use of benzodiazepines or antidepressants or neuroleptics.

||: Participants with impaired Clock drawing test.

**Figure 1:**
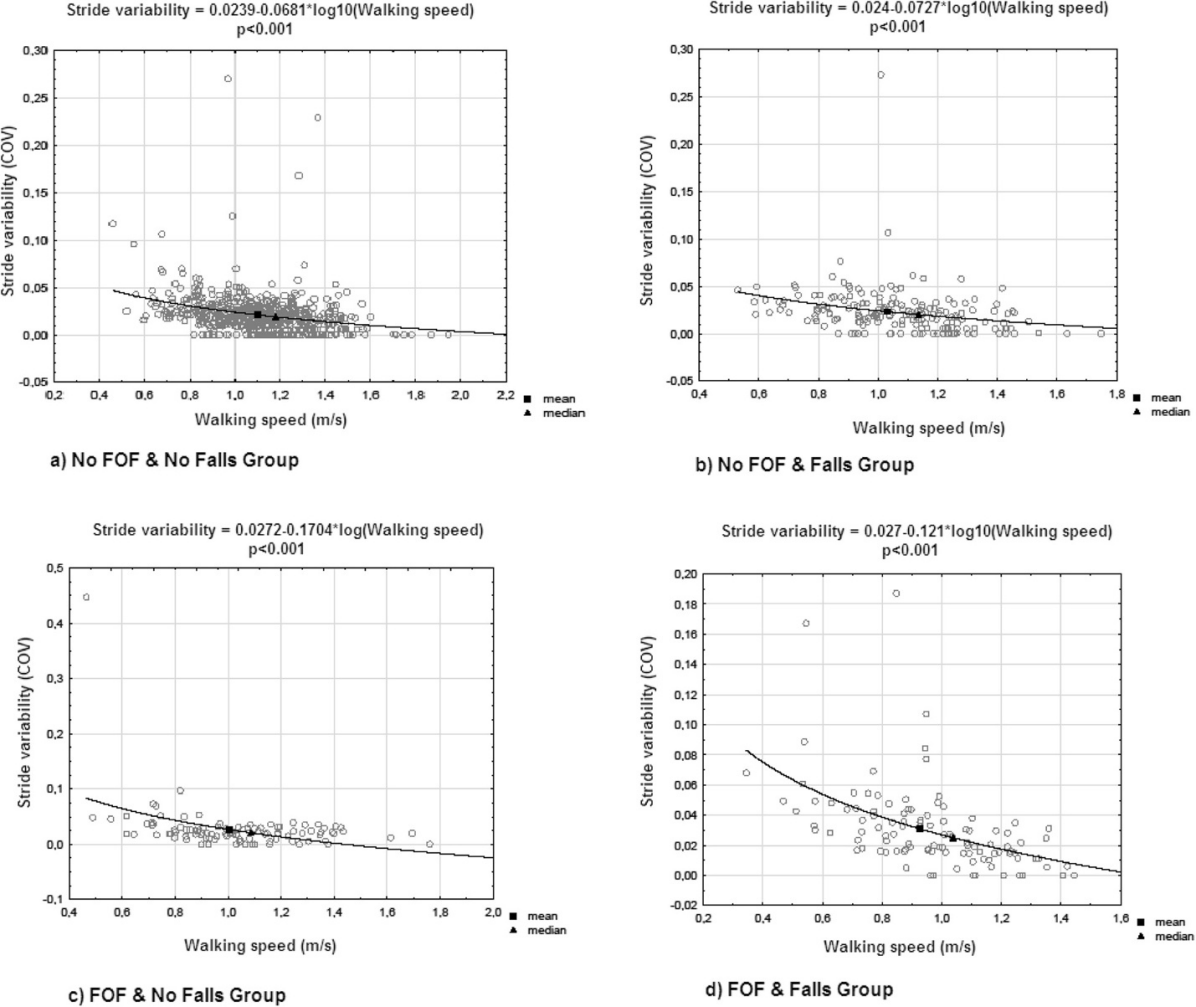
**Logarithmic regression of the association between stride variability and walking speed of participants separated into four groups based on fear of falling and history of falls (n = 1,023).** FOF: fear of falling.

Table 2 presents results from linear regression models investigating the association between CoV of STV and FOF. The univariate model shows that FOF with or without history of previous falls was associated with higher STV (P < 0.040). Age was positively associated with increase in STV when waking speed was not used as a covariable in the multiple regression models (P < 0.003). Gender, number of drugs daily taken per day, BMI, lower-limb proprioception, visual acuity, use of psychoactive drugs and cognitive impairment were not associated with STV. Adjustment on age, female gender, number of drugs daily taken per day, BMI, lower limb proprioception and visual acuity showed that only the combination of FOF with history of previous falls was associated to an increased STV (P < 0.015). Further adjustment on walking speed, as well as on cognitive parameters (i.e., use of psychoactive drugs, and cognitive impairment) made the association non significant. The adjusted R-squared for all models were low but increased with the adjustment on covariables (R^2^ = 0.011 for model 1, R^2^ = 0.026 for model 2, R^2^ = 0.028 for model 3, R^2^ = 0.043 for models 4 and 5).The association between STV and walking speed for the four groups of participants was assessed using a logarithmic regression (Figure 1). The mean and the median of STV for each group of participants were calculated. Results showed that lower walking speed value were associated to an increase in STV (P < 0.001). STV decreased when walking speed was: >1.18 m/s in the group of participants with no FOF and no falls; >1.14 m/s in those with no FOF and falls; >1.09 m/s in those with FOF and no falls; and >1.04 m/s in those with FOF and falls.

**Table 2:** **Multiple linear regression models showing the association between stride-to-stride variability of stride time (dependent variable) and fear of falling (independent variable, with the group with no fear of falling and no falls used as reference) adjusted on participants’ characteristics (n = 1023)**

	Change in CoV of stride time*														
	Model 1^†^			Model 2^†^			Model 3^†^			Model 4^†^			Model 5^†^		
	ß	95% CI	P-value	ß	95% CI	P-value	ß	95% CI	P-value	ß	95% CI	P-value	ß	95% CI	P-value
Fear of falling and falls combination															
No fear of falling and no falls	Ref			Ref			Ref				Ref		Ref		
No fear of falling and falls	0.017	[-0.003;0.005]	0.586	0.010	[-0.004;0.005]	0.747	0.006	[-0.004;0.005]	0.847	-0.006	[-0.004;0.004]	0.847	-0.005	[-0.004;0.004]	0.876
Fear of falling and no falls	0.066	[0.000;0.011]	0.038	0.056	[-0.001;0.010]	0.084	0.047	[-0.001;0.010]	0.142	0.030	[-0.003;0.008]	0.340	0.030	[-0.003;0.008]	0.339
Fear of falling and falls	0.110	[0.004;0.015]	0.001	0.096	[0.003;0.014]	0.003	0.082	[0.001;0.012]	0.014	0.044	[-0.002;0.009]	0.175	0.042	[-0.002;0.009]	0.192
Age				0.118	[0.000;0.001]	<0.001	0.106	[0.000;0.001]	0.002	0.055	[0.000;0.001]	0.094	0.047	[0.000;0.001]	0.157
Female gender				0.041	[-0.001;0.005]	0.205	0.044	[-0.001;0.006]	0.184	0.027	[-0.002;0.005]	0.401	0.030	[-0.002;0.005]	0.349
Number of drugs daily taken per day							0.049	[0.000;0.001]	0.136	0.004	[-0.001;0.001]	0.903	0.002	[-0.001;0.001]	0.941
Body mass index							0.052	[0.000;0.001]	0.100	-0.004	[0.000;0.000]	0.909	-0.006	[0.000;0.000]	0.837
Lower limb proprioception‡							-0.001	[-0.001;0.001]	0.960	0.016	[-0.001;0.001]	0.612	0.015	[-0.001;0.001]	0.636
Visual acuity§							-0.017	[-0.001;0.001]	0.606	0.015	[-0.001;0.001]	0.629	-0.021	[0.000;0.001]	0.500
Walking speed										-0,298	[-0,042;-0,027]	<0.001	-0.292	[-0.041;-0.026]	<0.001
Use of psychoactive drugs||													-0.016	[-0.002;-0.001]	0.608
Cognitive impairment#													-0.057	[-0.008;0.000]	0.060

CI = confident interval; CoV: Coefficient of variation; β: Coefficient of regression beta corresponding to increase or decrease in CoV of stride time expressed in%; *: increase or decrease; †: Separated models (Model 1: univariate model; Model 2: Model adjusted for age and gender; Model 3: Model 2 plus adjustment on number of drug daily taken, body mass index, lower limb proprioception and visual acuity; Model 4: Model 3 plus adjustment on walking speed; Model 5: Model 4 plus adjustment on use of psychoactive drugs and cognitive impairment); ‡: Mean value of left and right side and based on graduated tuning fork placed on the lower limb; §: Binocular visual acuity at a distance of 5 m with a Snellen letter test chart; ||: Use of benzodiazepines or antidepressants or neuroleptics; #: Participants with impaired Clock drawing test.

Finally, and as shown in Table 3, while using a walking speed ≥1.14 m/s (i.e., level of walking speed that did not influence STV) - which corresponds to the median of STV for all participants - the fully adjusted linear regression models showed that only the age and the combination of FOF with history of falls were associated with higher STV (P < 0.045), and being above this walking speed threshold was associated with a decrease in STV (P < 0.001). Adjusted R^2^ for models 1 and 2 were low, respectively at 0.052 and 0.051.

**Table 3:** **Multiple linear regression models exploring the association between stride-to-stride variability of stride time (dependent variable) and fear of falling (independent variable, with the group with no fear of falling and no falls used as reference) using a threshold for walking speed value (n = 1023)**

	Change in CoV of stride time*					
		Model 1^†^		Model 2^†^		
	ß	95% CI	P-value	ß	95% CI	P-value
Fear of falling and falls combination						
No fear of falling and no falls		Ref		Ref		
No fear of falling and falls	0.002	[-0.029;0.033]	0.955	0.003	[-0.028;0.035]	0.917
Fear of falling and no falls	0.039	[0.007;0.071]	0.219	0.041	[0.008;0.073]	0.206
Fear of falling and falls	0.067	[0.034;0.099]	0.043	0.066	[0.033;0.099]	0.044
Age	0.088	[0.054;0.122]	0.009	0.084	[0.050;0.118]	0.013
Female gender	0.042	[0.009;0.075]	0.201	0.042	[0.009;0.075]	0.201
Number of drugs daily taken per day	0.033	[0.001;0.066]	0.296	0.033	[0.001;0.066]	0.309
Body mass index	0.030	[-0.001;0.061]	0.338	0.030	[-0.002;0.061]	0.347
Lower limb proprioception‡	0.006	[-0.026;0.038]	0.855	0.004	[-0.028;0.037]	0.898
Visual acuity§	-0.003	[-0.035;0.029]	0.923	-0.004	[-0.036;0.029]	0.913
Walking speed ≥ 1.14 m/s	-0,165	[-0,196;-0,132]	<0.001	-0.164	[-0.196;-0.132]	<0.001
Use of psychoactive drugs||				-0.026	[-0.057;0.005]	0.397
Cognitive impairment#				-0.001	[-0.033;0.031]	0.981

CI = confident interval; CoV: Coefficient of variation; β: Coefficient of regression beta corresponding to change in CoV of stride expressed in%; *: increase or decrease; †: Separated models (Model 1: Model adjusted for age, gender, number of drug daily taken, body mass index, lower limb proprioception, visual acuity and walking speed ≥ 1.14 m/s; Model 2: Model 1 plus adjustment on use of psychoactive drugs and cognitive impairment); ‡: Mean value of left and right side and based on graduated tuning fork placed on the lower limb; §: Binocular visual acuity at a distance of 5 m with a Snellen letter test chart; ||: Use of benzodiazepines or antidepressants or neuroleptics; #: Participants with impaired Clock drawing test.

## Discussion

This study provides original information on the association of FOF with STV among older community-dwellers, highlighting that divergences previously reported on this association in the literature[2, 5, 8, 9] and in the present study mainly depend on the history of falls and the walking speed level. The latter parameter appears to play a major confounding role with a discontinued effect. This result is in concordance with the last published study on this topic, which underscored the hypothesis that walking speed could be a confounder in the relationship between high STV and FOF[5]. Indeed, the association between STV and FOF was no longer significant after adjustment on walking speed in the present study. Our study provides more information on this specific effect of walking speed. The combination of FOF with history of falls was associated with an increase in STV only for a walking speed above 1.14 m/s. These results suggest that at lower walking speed, STV mainly depends on a biomechanics effect that overrides the effects of all other covariables and thus prevents exploring the association of STV with FOF.

It has been previously reported that history of falls and FOF were independently related to an increase in STV[11]. Our study underscores a new insight, which corresponds to a synergistic effect when FOF and falls are combined. Indeed, taken alone they did not increase significantly STV and the magnitude of effect was very low, but together they highly increased STV. This result is in concordance with Maki et al.[22] who suggest this kind of interaction between FOF and falls. On the other hand, it has been previously reported that increased gait variability was a biomarker of falls[22]. Numerous studies reported that fallers compared to non-fallers had higher gait variability[9–22]. In addition, it has been shown that higher gait variability may predict falls[22]. First, the divergence with our results may be explained by the fact that we used a cross-sectional design, which is not best design to examine the causal relationship between two variables compared to cohort study design. Second, there could be a recall bias for falls assessment because it is well-known that a high proportion may forget the occurrence of falls, especially while the information on falls is retrospectively recorded compared to prospective collection of the event[23]. Third, we used STV while other studies found association between higher variability of other spatial-temporal gait parameters and falls[5]. It has been reported that there is a variation of the level of gait variability related to type of spatial-temporal gait parameter considered in healthy older adults with safe gait, the lowest value being reported with stride time and the highest with stride width[10].

The second main result of our study was the fact that the effect of the combination of FOF with falls on STV depended on the level of walking speed. A significant association was reported only for faster walking speed (e.g., above 1.14 m/s). In contrast, for lower walking speed, the effect of combination of FOF with falls disappeared, and the increase in STV was related only to the decrease in walking speed. This effect is a well-known biomechanics effect. For instance, Beauchet et al.[24] previously showed, while using a dual-task paradigm among healthy young adults, that the increase in STV was explained by the decrease in walking speed rather than an attention interference. This biomechanics effect was first underscored by Heiderscheit[25] who showed that stride time variability was greater at very slow stride velocity (between 0.2 m.s^-1^ and 0.6 m.s^-1^) as compared with speeds ranging from 0.8 to 1.4 m.s^-1^ in a sample of older adults. Our results are similar with a higher threshold value. One explanation could be related to the age and the relatively good health of the studied population. It is important to note that the threshold walking speed of 1.14 m/s reported in participants with FOF and history of falls is close to the walking speed required for safely crossing street traffic signals in a community, 1.1 m/s[26]. It may be suggested that for this group of individuals if their self-selected walking speed is above this threshold, the significant inverse association of walking speed and STV probably indicated their need to change their natural stepping rhythm in order to maintain dynamic postural stability. Whereas for those with a slower walking speed (i.e., under 1.14 m/s), the lack of significant association probably indicated that inability to adapt adequately their gait patterns. The clinical implication could be that the screening of walking speed is essential in this group of individuals[27]. If their walking speed is above the threshold speed, then the screening of STV may also be necessary and in case of abnormalities early intervention could be properly initiated.

A significant increase in STV related to the combination of FOF and falls at higher walking speed may be interpreted as a biomarker of the impairment of higher-level gait control. In terms of motor control, lower variability reflects an automatic process requiring minimal attention, whereas higher variability is related to major attention involvement[10]. Dual task-related gait changes, which are used to study the involvement of attention in gait control, have highlighted that the control of spatial-temporal stride parameters may differ from one parameter to another. For instance, it was shown that healthy younger adults devoted attention to balance control under dual task conditions, whereas the control of the walking-related rhythmic stepping mechanism did not change[28]. Both stride time and stride length variability are related to the control of the rhythmic stepping mechanism[29]. Lower variability values reflect the reliability of lower limb movements and the automated regular rhythmic feature of gait and are associated with safe gait[22]. Thus, an increase in STV due to the combination FOF with falls may be a biomarker of the impairment of higher levels of gait control.

Our study has a number of strengths. First, it is the largest population based study in older adults that examined the association of FOF with STV. Second, compared to previous published studies, the major potential confounders in our study were taken into account, particularly walking speed. Third, all participants had a comprehensive clinical examination and specific gait assessment with the GAITRite® system, which is a validated portable gait analysis system that allows simple objective gait measurements.

### Study limitations

There were also some methodological limitations in our study. First, the cross-sectional design used in the current original study is not the most adapted to examine the association of FOF with STV compared to a prospective cohort study design. Second, FOF was recorded only with a simple question. Although this assessment of FOF is validated[30], a questionnaire with several questions would prove better and provide more information on the level of FOF associated with STV. Third, although we were able to control for many characteristics likely to modify the association between FOF and STV, residual potential confounders might still be present in our study. For instance, it could be suspected that individual’s physical activity level may influence STV. There is only few published data on the latter point. In 2005, we provided the first evidence while comparing stride time variability under dual-task condition between 10 healthy community dwelling older adults with long-term practice of Jaques-Dalcroze eurhythmics and 11 healthy controls without any particular exercise routine[31]. No significant increase of stride time variability was found in the Dalcroze group with the interfering task of backward counting. In contrast, the healthy older subjects of the control group significantly increased their gait variability under dual task. In addition, all values of R-squared were small underlining that there was a large portion of variance of STV that was not accounted for confounders used in linear regression models. These low R-squared values also indicated that the strength of association was low between STV and tested dependent variables.

## Conclusions

The current cross-sectional study shows that the combination of FOF with falls is significantly associated with higher STV in community-dwelling older adults. This association depends on the level of walking speed, the increase in STV at lower walking speed being mainly explained by a biomechanics effect overriding FOF-related effects.

## Authors’ original submitted files for images

Below are the links to the authors’ original submitted files for images. Authors’ original file for figure 1

## Abbreviations

BMI: Body mass index
CoV: Coefficient of variation
FOF: Fear of falling
STV: Stride-to-stride variability of stride time.
