# Supplementary figures and images for: The influence of fear of falling on gait variability: results from a large elderly population-based cross-sectional study
Source: J Neuroeng Rehabil. 2014 Aug 29;11:128. doi: 10.1186/1743-0003-11-128 (PMC4156618; doi:10.1186/1743-0003-11-128)

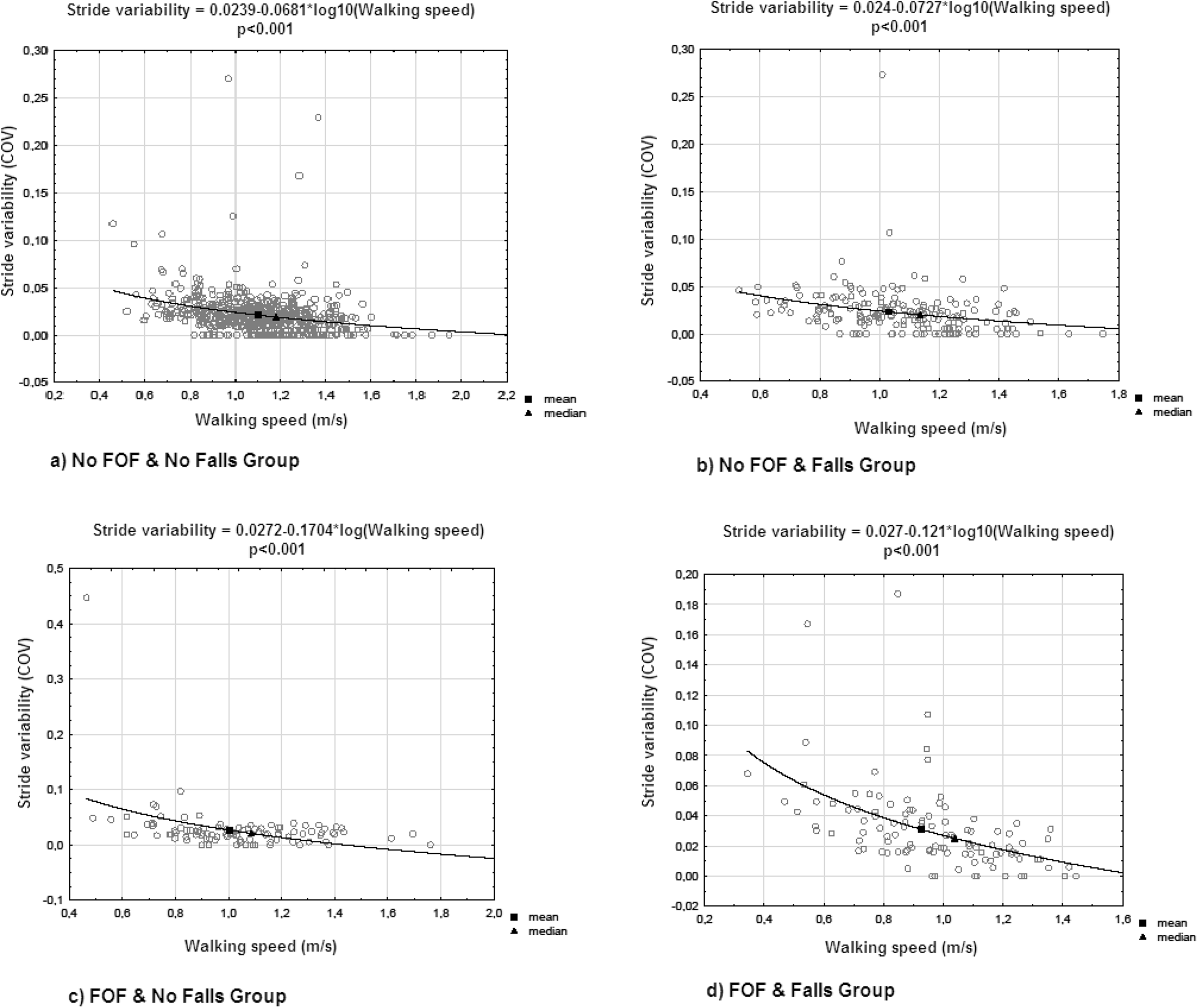

Supplement: Supplementary file 1 — Authors’ original file for figure 1 [file 12984_2014_650_MOESM1_ESM.tif]
